# Insights into the role of Notch signalling in cilia motility regulation

**DOI:** 10.1186/2046-2530-4-S1-P81

**Published:** 2015-07-13

**Authors:** P Sampaio, B Tavares, R Jacinto, S Lopes

**Affiliations:** 1 Cilia Regulation and Disease Group, CEDOC - Chronic Diseases Research Center, Lisboa, Portugal

## Background

Our previous work has demonstrated that Notch signalling modulates cilia length in the zebrafish left-right organizer (LRO) [1]. However, we also found that the axonemal motor Dnah7, an inner dynein arm motor protein, is upregulated in Notch signalling mutants where motile cilia number is exacerbated related to immotile cilia [1]. Moreover, the knock-down of this motor protein drastically affected cilia motility in the LRO, blocking the nodal flow.

## Objective

To link Notch signalling to the regulation of cilia motility genes that may be upstream of *dnah7*.

## Methods

Gene expression of potential motility-mediator targets were validated by qPCR in deltaD mutants. Whole mount in situ hybridization was performed to assess dnah7 gene expression in zebrafish embryos at various stages of development. We used high-speed videomicroscopy to study olfactory pit and LRO cilia motility in dnah7 zebrafish morphants. The ultrastructure of static cilia was analysed from embryos injected with dnah7-MO.

## Results

Dnah7 is upregulated in deltaD mutants. Dnah7 is expressed in other organs with motile cilia, such as the olfactory pits, brain ventricles and pronephros. Down-regulation of dnah7 showed also to affect motility in olfactory pits and pronephros. We are validating microarray data by qPCR and will present a model on how Notch signalling may affect the expression of *dnah7*.

## Conclusion

Dnah7 knock-down affects cilia motility in various ciliated organs during zebrafish development. We found specific target genes from the foxj1 and rfx families, which encode regulatory factors that may be involved in a Notch signalling transduction pathway linked to cilia motility.

Supported by FCT-ANR/BEX-BID/0153/2012 grant.
